# Segmented Polyurethanes Based on Adipate and Sebacate Biodegradable Polyesters for Use as Nerve Guide Conduits in Peripheral Nerve Regeneration

**DOI:** 10.3390/polym17121692

**Published:** 2025-06-18

**Authors:** Alexis B. Sabido-Barahona, Rossana F. Vargas-Coronado, Fernando Hernández-Sánchez, Antonio Martínez-Richa, José L. Gómez Ribelles, Juan V. Cauich-Rodríguez, Angel Marcos-Fernández

**Affiliations:** 1 Materials Department, Centro de Investigación Científica de Yucatán, A. C., Calle 43 No. 130 x 32 y 34, Col. Chuburná de Hidalgo, Merida 97205, Yucatan, Mexico; alexis.sabido@estudiantes.cicy.mx (A.B.S.-B.); ross@cicy.mx (R.F.V.-C.); fhs@cicy.mx (F.H.-S.); 2 Departamento de Química, Universidad de Guanajuato, Noria Alta s/n, Guanajuato 36050, Guanajuato, Mexico; richa@ugto.mx; 3 Centre for Biomaterials and Tissue Engineering, CBIT, Universitat Politècnica de València, 46022 Valencia, Spain; jlgomez@ter.upv.es; 4 Centro de Investigación Biomédica en Red de Bioingeniería Biomateriales y Nanomedicina (CIBER-BBN), Instituto de Salud Carlos III, Av. Monforte de Lemos, 3-5. Pabellón 11. Planta 0, 28029 Madrid, Spain; 5 Institute of Polymer Science and Technology (ICTP-CSIC), Juan de la Cierva, 3, 28006 Madrid, Spain

**Keywords:** segmented polyurethanes, nerve conduits, nerve regeneration materials, biodegradable polyurethanes, polyurethane characterization, hard and soft segments, phase separation

## Abstract

This study investigated the chemical, thermal, and mechanical properties of segmented polyurethanes (SPUs) synthesized using less common biodegradable polyester polyols, specifically poly(adipate) (PAD) and poly(sebacate) (PSC), to evaluate their potential as nerve guidance conduits (NGCs) in peripheral nerve regeneration. The synthesis of novel 4,4′ methylene-bis-cyclohexyl diisocyanate (HMDI) SPUs was conducted in a two-step process: prepolymer formation and chain extension with 1,4-butanediol (BO) or 1,4-butanediamine (BA). SPUs were synthesized with two molar ratios—polyol:HMDI:BA/BO at 1:2:1 and 1:3:2 for the PAD:HMDI:BA system—to optimize mechanical properties. 1HRMN analysis verified the expected chemical structure of SPUs, whereas Raman and IR spectroscopy confirmed successful polyurethane synthesis. X-ray diffractograms showed that PAD-based SPUs (SPUPAD) were amorphous while PSC-based SPUs (SPUPSC) exhibited semi-crystalline behavior. SPUPAD showed only one degradation stage by TGA, while DSC showed one thermal event. In contrast, SPUPSC exhibited two degradation stages and three thermal events that confirmed phase separation. The longitudinal tensile properties of an NGC fabricated from SPUA-PAD-2 (PAD:HMDI:BA (1:3:2)) after 30 days of immersion in water (25 °C) showed a lower modulus (4.46 ± 0.5 MPa) than native intact nerves (15.87 ± 2.21 MPa) but a similar modulus to extracted nerves (8.19 ± 7.27 MPa). This system exhibited a longitudinal tensile force of 11.1 ± 1.6 N, which is lower than that of peripheral nerves (19.85 ± 7.21 N) but higher than that of commercial collagen-based nerve guide conduits (6.89 ± 2.6 N). The observed properties suggest that PUA-PAD-2 has potential as a biomaterial for nerve regeneration applications.

## 1. Introduction

The peripheral nervous system (PNS) is a well-structured network of tissues that connects the central nervous system to various organs throughout the body [1]. Among the different types of peripheral nerve injuries, neurotmesis is the most severe, characterized by a complete nerve gap and a loss of both sensory and motor function. Peripheral nerve repair presents significant surgical challenges, with a high incidence in Europe and the USA, exceeding 100,000 cases of neurotmesis annually [2]. The gold standard for peripheral nerve repair is autograft transplantation suitable for nerve gaps smaller than 50 mm. However, autograft use is limited due to several factors including low availability, donor site morbidity, and size mismatch. Allografts are an alternative but require immunosuppression and carry the risk of infection [3,4].

When autografts and allografts are not feasible, nerve guidance conduits (NGCs) become the preferred treatment option. NGCs are tubular structures surgically implanted to bridge the gap between the severed nerve ends. They offer several benefits including the protection of nerves from external tissues, guide axonal growth, and provide the necessary environment for nerve regeneration. Early NGCs were primarily composed of silicone (PDMS), but their clinical application is limited due to their non-biodegradable nature, which can lead to nerve compression [5]. Several FDA-approved NGCs are currently available on the market, including Neuragen^®^ (collagen), Reaxon Plus (chitosan), Neurolac (PLDL), SaluBridge/SaluTunnel (PVA), NeuroTube/Nerbridge (PGA), and Axoguard (allograft) [6]. Despite this variety, definitive clinical evidence to determine the optimal NGC for nerve regeneration remains elusive. Consensus suggests that NGCs are suitable for repairing peripheral nerve defects up to 30 mm in length [7]. A crucial characteristic of any NGC is its dimensions, as the conduit must accurately match the length of the nerve gap and the internal diameter of the injured nerve to facilitate successful regeneration. Additionally, NGCs must be biocompatible with an appropriate degradation rate and mechanically stable to withstand the loads from physiological movements [8,9]. Recent research has explored the use of natural, synthetic, and hybrid materials to enhance the regenerative capacity of neural guidance channels. Natural polymers such as chitosan, hyaluronic acid, collagen, and silk have been widely studied for the manufacture of NGCs due to their biocompatibility, but they often suffer from high degradation rates and low mechanical properties [10]. On the other hand, synthetic polymers such as polylactic acid (PLA), polycaprolactone (PCL), polyglycolic acid (PGA), and polylactic-co-glycolic acid (PLGA) offer superior mechanical properties and are more easily processed [11]. Moreover, efforts are underway to develop biodegradable NGC versions based on these polymers to mitigate the risk of nerve compression. By combining the advantages of both natural and synthetic materials, hybrid approaches aim to create NGCs with optimal biocompatibility, mechanical support, and controlled degradation profiles. Polyurethanes (PUs) represent another promising material for the fabrication of neural guidance channels (NGCs). While widely utilized in vascular grafts and drug delivery systems, their application [12,13] in NGCs remains relatively unexplored. PUs are typically synthesized as segmented polyurethanes (SPUs) through a two-step polycondensation process [14]. This results in a material with distinct soft and hard segments. The soft segment, formed by reacting a polyol (e.g., polyester) with excess diisocyanate, contributes to material flexibility. The hard segment, created by further reaction with a chain extender, influences overall mechanical strength. The versatility of SPUs lies in their tunable properties. Through the modification of the composition of the soft and hard segments, it is possible to tailor their mechanical properties to match the specific requirements of peripheral nerve regeneration [15,16]. Furthermore, the choice of polyol significantly impacts the degradation rate and mechanical behavior of the material [17]. Biodegradable SPUs can be synthesized using various polyols, including polycaprolactone (PCL), polylactic acid (PLA), polyethylene glycol (PEG), and polypropylene glycol (PPG). Some studies have explored the use of PCL and PEG in combination to fine-tune the material’s properties, demonstrating that the relative proportions of these polyols can influence both mechanical strength and the degradation rate [18,19]. The selection of diisocyanate also plays a crucial role in determining the biocompatibility and degradation profile of the resulting SPU [20]. Cyclic or aliphatic diisocyanates, such as 4,4′-methylene-bis-cyclohexyldiisocyanate (HMDI), 1,4-butane diisocyanate (BDI), and 1,6-hexamethylene diisocyanate (HDI), are generally considered safer due to the non-toxic nature of their degradation products [21].

This study focuses on developing novel, biodegradable neural guidance channels (NGCs) using HMDI-based segmented polyurethanes (SPUs). For this, less common polyols like poly (ethylene glycol-butanediol-diethylene glycol adipate) (PAD) and an aliphatic polyester based on sebacic acid and 1,3-propanediol (PSC) were used as soft segments. In addition, either butanediol (BO) or butanediamine (BA) was used as a chain extender for further property tuning as it is generally accepted that ureas derived from the reaction of isocyanates with amines provide higher phase separation and better mechanical properties. Finally, the effect of the hard- and soft-segment ratios on the properties of the SPU was studied by varying the molar ratio of polyol, diisocyanate, and the chain extender. A comprehensive characterization was conducted through chemical, thermal, mechanical, and degradation analyses. Following this, NGCs were fabricated by the roto-evaporation method and their mechanical performance evaluated and compared with native peripheral nerves and some FDA-approved NGCs (Neuragen and NeuroTube). This research has the potential to contribute significantly to the advancement of nerve regeneration therapies by providing a platform for the development of improved and more effective neural guidance channels.

## 2. Materials and Methods

### 2.1. Materials

Segmented polyurethanes were synthesized using two different biodegradable polyols: poly (ethyleneglycol-butanediol-diethyleneglycol adipate) (PAD) (MW = 2000, Glypol 4027 from Condensia, Barcelona, Spain) and poly (1,3 propanediol sebacate) (PSC) (MW = 2300, supplied by Merquinsa—now Lubrizol—Barcelona, Spain). Other chemicals used in the synthesis were purchased from Sigma Aldrich including 4,4′-methylene-bis-cyclohexyldiisocyanate (HMDI), Tin (II) octanoate (Oct-Sn), 1,4-butanediamine (BA), 1,4-butanediol (BO), and dimethylformamide (DMF).

### 2.2. Segmented Polyurethane Synthesis

**Scheme 1:**
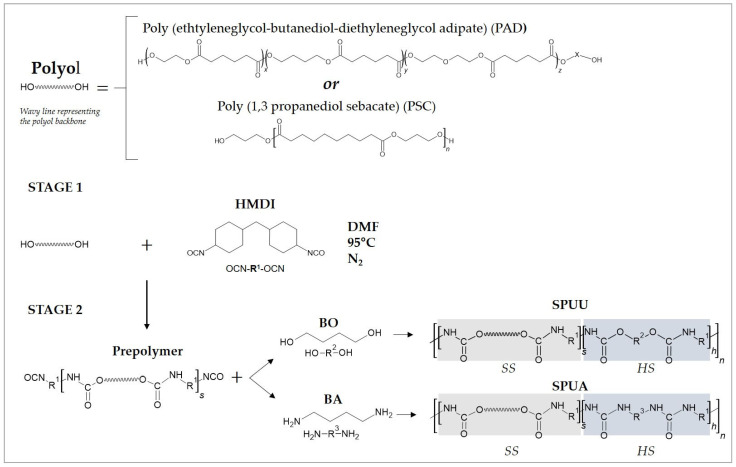
Synthesis of SPUU and SPUA based on PAD or PSC polyols.

Segmented polyurethane based on PAD (SPUPAD) or PSC (SPUPSC) was synthesized in two stages (Scheme 1): prepolymer formation and chain extension. Prepolymer formation was achieved after dissolving either PAD or PSC in DMF in a glass reactor (95 °C, nitrogen atmosphere, 120 rpm) followed by dropwise addition of HMDI in the presence of Oct-Sn, leaving the system to react for 4 h. For chain extension, either BO or BA was added to the reactor and allowed to react for 2 further hours. Finally, the product was precipitated in cold water and stirred for 24 h. The polymer obtained was recovered by filtration and drying at 60 °C under vacuum for 24 h. The initial molar ratio was 1:2:1 for polyol–HMDI–chain extender, but this was increased to a molar ratio of 1:3:2 for the system PAD:HMDI:BA for improved handling and mechanical performance. A non-segmented polyurethane (PU-PAD/PU-PSC) was synthesized with a polyol–HMDI molar ratio of 1:1 following the same procedure described for the first stage of segmented polyurethane synthesis. When either BO or BA was used as a chain extender, a segmented polyurethane–urethane (SPUU) or a segmented polyurethane–urea (SPUA) was obtained (see reaction scheme). Hard-segment content (HS%, effective HS) was calculated using the mass of isocyanate reacting with the chain extender ${(W}_{I})$ and the chain extender mass ${(W}_{\mathrm{CE}})$, according to Equation (1). Table 1 summarizes the different synthesized polymers with their corresponding soft-segment (SS) and hard-segment (HS) contents.

(1) $$\mathrm{HS}\;\left[\%\right]=\frac{W_{I}-W_{\mathrm{CE}}}{\mathrm{Total}\;\mathrm{mass}\;\mathrm{of}\;\mathrm{SPU}\;}\times \;100$$

### 2.3. SPU Film Preparation and Characterization

For subsequent characterizations, polyurethane films were prepared by dissolving 2 g of the polymer in 40 mL of tetrahydrofuran (THF). The resulting solution was poured onto a leveled Teflon mold and then covered with a conical glass funnel to facilitate slow solvent evaporation over 24 h. Finally, the obtained film was removed and stored in a desiccator.

#### 2.3.1. Spectroscopic and Structural Characterization

Fourier-transform infrared (FTIR) spectroscopic analysis was performed using a Thermo Scientific Nicolet 8700 in total reflectance mode, covering a wavenumber range from 4000 to 650 cm⁻¹, with a resolution of 4 cm⁻¹ and an average of 100 scans. Additionally, Raman spectra in the wavelength range of 4000–200 cm⁻¹ were collected using a Renishaw inVia Reflex spectrometer (Gloucestershire, UK), with a 633 nm laser source, 100% of power, and an exposure time of 10 s. In addition, the chemical structure of polyurethanes was determined by proton nuclear magnetic resonance (^1^H-NMR) by using 500 MHz Bruker Avance equipment (Billerica, MA, USA) with deuterated chloroform (CDCl_3_) dissolved samples. The elemental atomic percentage of SPUU/SPUA was analyzed using X-ray photoelectron spectroscopy (XPS) with a Thermo Scientific K-Alpha X-ray Photoelectron Spectrometer (Thermo Fisher Scientific, Waltham, MA, USA), equipped with a monochromatic Al Kα source (1486.6 eV). The samples were subjected to 30 s of argon (Ar) ion erosion. The pressure was reduced to 5 × 10^−9^ mbar, and static charge compensation was applied using an Ar ion beam. To estimate the contributions of the C1s, O1s, and N1s bonds, a deconvolution fit was applied to the peaks of each element using Gaussian functions. The crystalline structure of SPUU/SPUA films was determined using a Bruker D-8 X-ray diffractometer with a voltage, current, and step time of 40 V, 30 mA, and 0.5 s, respectively.

#### 2.3.2. Thermal Characterization

The degradation temperatures of PUs were obtained using a Perkin Elmer TGA-8000 in a temperature range of 50 °C to 650 °C, at a heating rate of 10 °C/min, under a nitrogen atmosphere. Differential scanning calorimetry (DSC-250 from TA Instruments, New Castle, DE, USA) was used to determine the glass transition (Tg) and melting temperature (Tm) of SPU and evaluate phase separation. For this, approximately 10 mg of the sample was heated from −90 °C to 200 °C at a heating rate of 10 °C/min in a nitrogen atmosphere.

#### 2.3.3. Accelerated Degradation

Degradation studies were carried out by immersing SPUA films (n = 3) in four different aqueous media: distilled water, 30% $H_{2}O_{2},$ 2M HCl, and 5N NaOH under reflux at 100 °C for 24 h. The remaining mass was recovered, dried at room temperature, and weighed. The percentage of degraded mass was determined by the initial mass ($W_{0}$) and remaining mass ($W_{r}$) following Equation (2). FTIR spectra of degraded materials were obtained and compared with non-degraded SPU.

(2) $$\mathrm{Degraded}\;\mathrm{mass}\;\left[\%\right]=\frac{W_{0}-W_{r}}{W_{0}}\;\times \;100$$

#### 2.3.4. Uniaxial Tension Test on Films

The elastic modulus (E), maximum strength ($\sigma_{\max}$), and maximum deformation ($\epsilon_{\max}$) of SPU films were obtained according to the ASTM-D882 Standard [22], using a Universal Testing Machine MiniShimadzu AGS-X with a 100 N load cell with a resolution of 0.1 N and a head travel speed of 50 mm/min. Dog bone-shaped films (15 × 5 mm), fitted with an adhesive tape on the top and bottom of the specimen to prevent slipping from the grips, were used for tensile experiments.

### 2.4. Conduit Fabrication by Roto-Evaporation

**Figure 1:**
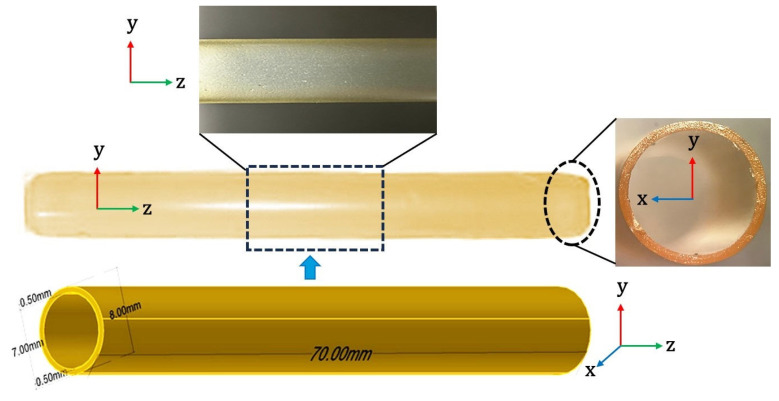
Dimensions of NGC manufactured by roto-evaporation method.

First, an SPU solution was prepared by adding 1.5 g of polymer to 10 mL of THF. A glass rod with a diameter of 7 mm was used as a mold and placed horizontally inside a glass tube containing the polymeric solution. The soaked mold was rotated at low speed (40–60 rpm) for approximately 30 s until the solvent evaporated. This procedure was repeated and the thickness measured with a Mitutoyo micrometer, until a wall thickness of 0.5 mm was reached. Finally, the SPU conduit was removed from the glass mold. The dimensions of SPU conduits were set at 70 mm length with an outer diameter of 8 mm, an inner diameter of 7 mm, and a wall thickness of 0.50 mm (see Figure 1). These dimensions are similar to the collagen nerve conduits manufactured by NeuraGen [23]. The outer diameter of the manufactured conduit is within the range reported for peripheral nerves (1–20 mm) [24].

#### Longitudinal and Radial Tension Tests on SPU Conduits (NGCs)

**Figure 2:**
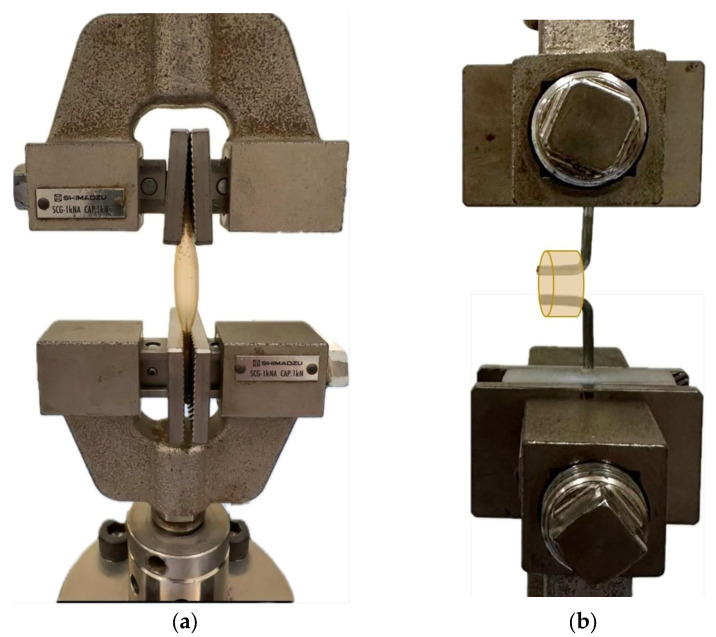
(**a**) Longitudinal and (**b**) radial tension test setup.

Currently, there is no ISO standard for obtaining the mechanical properties of nerve guide conduits; however, in this work, longitudinal and radial tension tests were performed according to ISO 7198:1998 [25] for vascular grafts. The Universal Testing Machine MiniShimadzu AGS-X with a 100 and 1000 N load cell and a head travel speed of 50 mm/min was used for the radial and longitudinal tests, respectively. These tests were carried out using only the NGC manufactured with SPUA-PAD-2. The longitudinal tension test was performed with SPU conduits positioned in grips with a distance of 3 cm between them (see Figure 2). The mechanical properties of tubular structures are typically reported through force–displacement curves, which facilitate the determination of load and maximum displacement. Moreover, in this study the stress–strain curve data was also calculated from force–displacement curves and the initial cross-sectional area of the tube to determine the values of E, $\sigma_{\max}$, and $\epsilon_{\max}$ of tubular conduits. The elastic moduli of SPUA conduits and SPU/SPUA films were compared to observe changes in mechanical properties. For the radial tension test, rings with a length of 10 mm ($L_{c}$) and internal diameter of 7 mm were used. These rings were placed radially and tested using two circular metal hooks with diameters of 2.4 mm, which were then secured into metal grips (see Figure 2b). The load–displacement curve data was obtained, and reported parameters include the maximum radial force ($F_{c\max}$) and the circumferential tensile strength ($r_{\max}$), defined according to Equation (3):

(3) $$r_{\max}\left[N/\mathrm{mm}\right]=F_{\mathrm{cmax}}\;/\;2L_{c}$$

## 3. Results and Discission

### 3.1. Spectroscopy Studies

**Figure 3:**
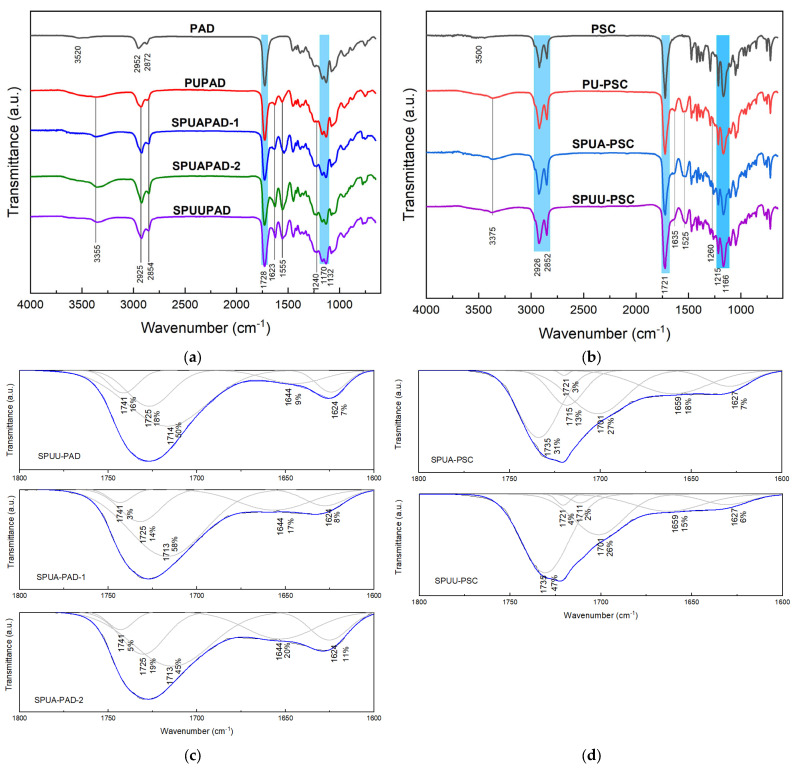
FTIR spectra of polyol/PU/SPUA/SPUU synthesized with (**a**) PAD and (**b**) PSC. Deconvolution in carbonyl region for PAD-based polyurethanes (**c**) and PSC-based polyurethanes (**d**).

Figure 3 shows the main IR peaks of pristine polyadipate (PAD) (cm^−1^): 3520 $\nu_{s}$ (OH), 2952 and 2872 $\nu_{\mathrm{as}}$ (${\mathrm{CH}}_{2}$), 1725 $\nu_{s}$ (C=O), and 1170 and 1132 $\nu_{\mathrm{as}}$ (C-O-C). These signals were also observed in the PU-PAD model polyurethane with additional bands located at (cm^−1^) 3355 $\nu_{s}$ (N-H), 1623 $\nu_{s}$ (C=O (amide I)), 1555 ($\nu_{s}\;$(C-N), $\delta_{s}$ (N-H, amide II)), and 1240 ($\nu_{s}\;$(C-N), $\delta_{s}$ (N-H), amide III), confirming the reaction between the diisocynate and the polyol [26]. In contrast, for SPUPAD the band at 1728 cm^−1^ broadens while the absorptions at 1623, 1555, and 1240 cm^−1^ are more intense due to the incorporation of a hard segment that contains urethane/urea groups when BO or BA was used. In the SPUAPAD-2 sample, peaks at 3355 cm^−1^ and 1623 cm^−1^ are more intense on the SPUPAD group, due to the higher amount of hard segments and the greater urea content. On the other hand, the main IR peaks of pristine PSC polyol were located at (cm^−1^) 3500 $\nu_{s}$ (OH), 2926 and 2852 $\nu_{\mathrm{as}}$ (${\mathrm{CH}}_{2}$), 1721 $\nu_{s}$ (C=O), and 1215 and 1166 $\nu_{\mathrm{as}}$ (C-O-C). The synthesis of PU-PSC polyurethane was confirmed with peaks located at (cm^−1^) 3375 $\nu_{s}$ (N-H), 1721 $\nu_{s}$ (C=O), 1635 $\nu_{s}$ (C=O, amide I), 1525 ($\nu_{s}$ (C-N) and $\delta_{s}$ (N-H, amide II)), and 1260 cm^−1^ ($\nu_{s}$ (C-N), $\delta_{s}$ (N-H), amide III). Similarly to the polymers synthesized with PAD, in SPUPSC the intensity in urethane bands at 3375 cm^−1^ and 1635 cm^−1^ increased due to the incorporation of the hard segment. The increase in urea content (overlapped in 1635 cm^−1^) was observed not only in BA-based polymers but also in BO-based ones, possibly due to the presence of water. Furthermore, the presence of the isocyanate-group signal (2260 cm^−1^) was not observed in any sample, indicating that the reaction was completed [27].

The carbonyl region (C=O) of the FTIR spectrum (1800–1600 cm^−1^) for polyurethanes was deconvoluted in order to evaluate their structural organization and microphase separation (Figure 3c,d). For SPUA-PAD up to five peaks were identified. The band at 1624 cm^−1^ is related to hydrogen-bonded and disordered urea carbonyl group [28]. The signal at 1644 cm^−1^ corresponds to hydrogen-bonded and ordered urea carbonyl, and its intensity depends on the hard-segment content. The absence of absorption at 1690 cm^−1^ indicates no free urea groups. The deconvolution of the IR spectrum of SPUs based on PAD showed the absence of a signal at 1701 cm^−1^ and the presence of a band at 1713 cm^−1^, suggesting a hydrogen-bonded urethane in the soft phase or the lack of a well-defined hard segment. Additionally, absorptions at 1725 cm^−1^ can be assigned to PAD ester. Finally, the low intensity at 1741 cm^−1^ indicates a small amount of free urethane carbonyl groups, suggesting that the phases are mixing through hydrogen bonding. Meanwhile, the increase in hard content from SPUA-PAD-1 (13.4%) to SPUA-PAD-2 (23.6%) resulted in a more intense urea carbonyl peak at 1644 cm^−1^.

In the case of SPUs based on PSC, five peaks were also detected in the carbonyl region. The presence of signals at 1701–1703 cm^−1^ indicates hydrogen-bonded carbonyl urethanes in the hard segment [29], at 1711–1715 cm^−1^ indicates hydrogen-bonded urethanes in the soft segment, and at 1735 cm^−1^ indicates free urethane carbonyls. Also, the band observed at 1659 cm^−1^ indicates disordered urea’s hard microphase.

**Figure 4:**
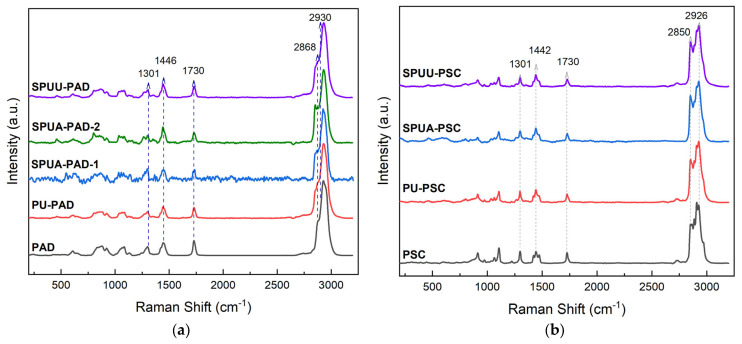
Raman spectra of polyol/PU/SPUA/SPUU synthesized with (**a**) PAD and (**b**) PSC.

The Raman spectra of PAD and PSC polyurethanes (Figure 4) support the functional groups identified in IR. For SPUPAD, the main peaks are located at (cm^−1^) 2930 (asymmetric CH₂), 2868 (symmetric CH₂), 1730 (C=O in polyol and C=O in amide II for urethane bond), 1446 (CH₂), and 1301 (CH₂). For SPUPSC (Figure 4b) the corresponding peaks are located at 2926, 2850, 1730, 1442, and 1301 cm^−1^.

**Figure 5:**
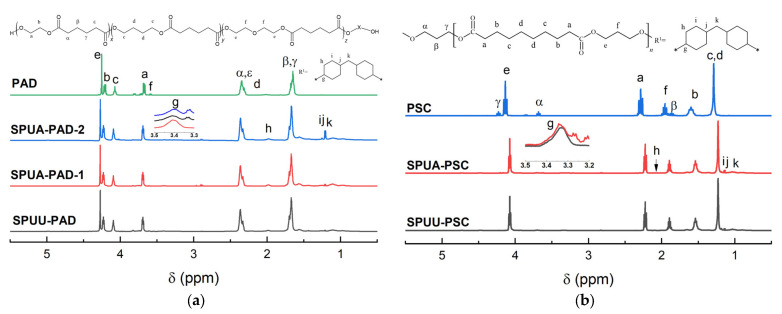
^1^H NMR spectrum of pristine (**a**) PAD, SPUPAD, (**b**) PSC, and SPUPSC.

**Figure 6:**
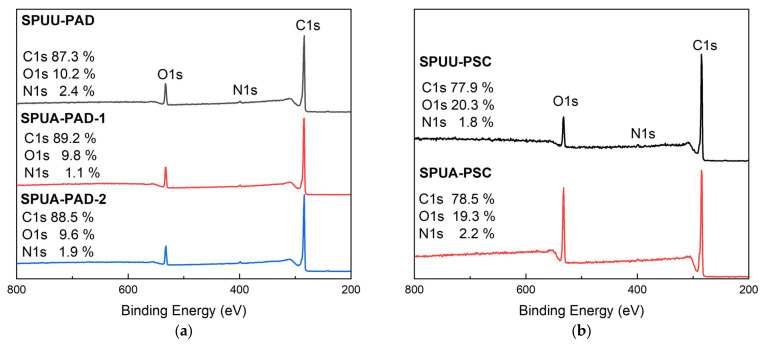

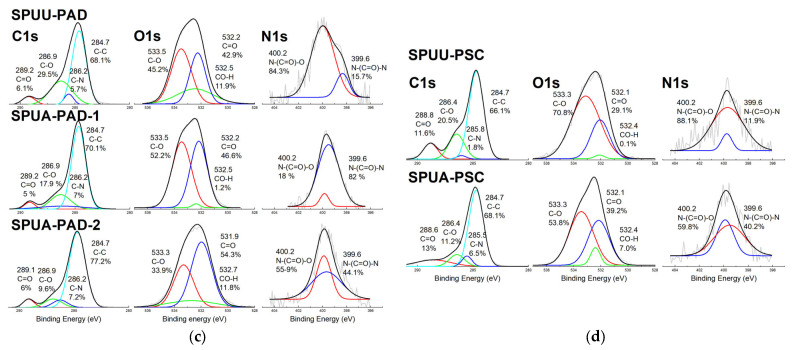
(**a**,**b**) XPS survey spectra and (**c**,**d**) deconvolution of C1s, N1s, and O1s scans of SPUU/SPUA synthesized with PAD and PSC.

^1^H NMR analysis (Figure 5) shows characteristic peaks of both polyols, with these being the major component in segmented polyurethanes. In SPUPAD spectra, this polyol was identified with shifts at δ(ppm) 4.27 (e), 4.22 (b), 4.09 (c), 3.7 (a, b), 3.6 (f), 2.36 (α, ε), 2.32 (d), and 1.66 (β, γ). The proton signal of the NH group has been reported at 3.32 ppm (g) (see inset of Figure 5 and Figure 6), confirming the formation of urethane/urea bonds [30]. Additionally, HMDI signals were found at 1.96 (h) and 1.2–1.5 (i, j, k). On the other hand, the signals for PSC are located at δ(ppm) 4.23 (γ), 4.07 (e), 3.68 (α), 2.22 (a), 1.89 (f), 1.80 (β), 1.53 (b), and 1.23 (c, d). Proton signals of NH in SPUPSC exhibit several similarities with SPUPAD. It is interesting to note that the peaks at 1.2–1.5 ppm (i, j, k), originating from HMDI, are more intense in the SPUA-PAD-2 sample. This is directly related to the hard-segment percentage of 23.6%, which is the highest in the segmented polyurethane synthesized.

The XPS spectra of segmented polyurethanes made with PAD and PSC are shown in Figure 6. The peaks indicate the presence of three main elements—C1s, N1s, and O1s—with a binding energy range of 291–282, 405–396, and 537–528 eV, respectively. Figure 6a,b show the XPS survey spectra, while Figure 6c,d show the peak deconvolution of C1s, N1s, and O1s peaks of synthesized segmented polyurethanes and polyurethanes–ureas.

The SPUU-PAD sample had the highest amount of O1s bonds (10.2%), and the deconvolution of the C1s peak indicated that C–O and C=O bonds are present in greater amounts; both findings suggest the highest percentage of urethane groups due to the BO chain extender. In contrast, the amount of N1s increased from 1.1 to 1.9% for SPUA-PAD-1 and SPUA-PAD-2, respectively, and the percentage of N–(C=O)–O bonds rose from 18% to 55.9%, indicating an increase in the hard-segment percentage.

On the other hand, SPUPSC samples contained the highest percentages of O1s (20.3% and 19.3%) among all segmented polyurethanes synthesized due to the higher molecular weight of PSC. It is interesting to observe that for SPUA-PSC, there is a higher percentage of C–N and N–(C=O)–N bonds (6.5% and 40.2%, respectively) compared to SPUU-PSC, with 1.8% and 11.9%, respectively. This is due to the urea group present in its hard segment caused by the BA chain extender.

### 3.2. Thermal Studies

**Figure 7:**
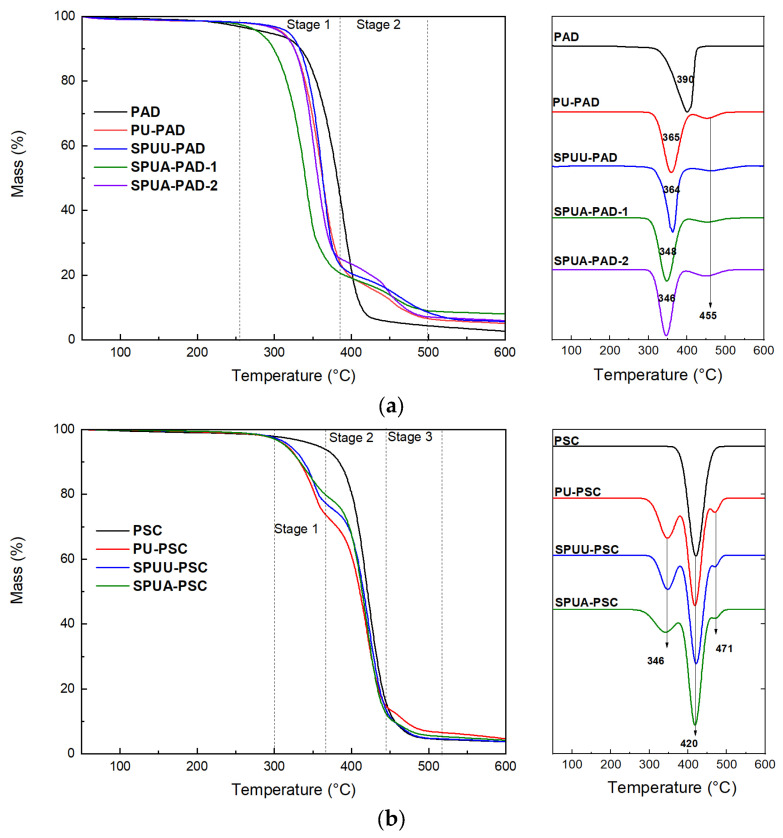
TGA and DTGA thermograms of polyol/PU/SPUU/SPUA synthesized with (**a**) PAD and (**b**) PSC.

Thermogravimetric thermogram (TGA) curves and derivative thermogravimetric (DTGA) curves of polyols and polyurethanes are shown in Figure 7. From the TGA curves it is observed that PUs made from PAD and PSC started their degradation at lower temperatures compared to the polyol used in their synthesis. The initial decomposition (stage 1) for segmented polyurethanes is sometimes attributed to the degradation of the hard segment. However, for SPUPAD, this behavior was not observed, likely due to the mixing of hard and soft segments. This mixing resulted in a reduced thermal stability of polyurethane, which decreased further as the hard-segment content increased, as depicted for SPUPAD samples. Additionally, in the DTGA curve for PAD polyurethanes, degradation peaks shifted to lower temperatures (346–365 °C) compared to the polyol (390 °C). In contrast, PSC polyurethanes exhibited hard-segment decomposition in three stages, where the mass loss in stage 1 corresponded to approximately 13.7% (a value close to the 11.9% hard-segment percentage calculated by stoichiometry and described in Table 1) and a maximum DTGA peak at 346 °C. The second decomposition temperature (stage 2) indicated thermal degradation of the soft segments of polyurethanes with maximum degradation at 420 °C, similarly to the pristine PSC. Some authors indicated that when a DTGA peak is a superposition of pure components, it may suggest incompatibility [31], but in SPUPSC, it could be a result of a phase separation between soft and hard segments. The final stages of degradation showed peaks at 455 and 471 °C for SPUPAD (stage 2) and SPUPSC (stage 3), respectively, indicating the degradation of char residues.

**Figure 8:**
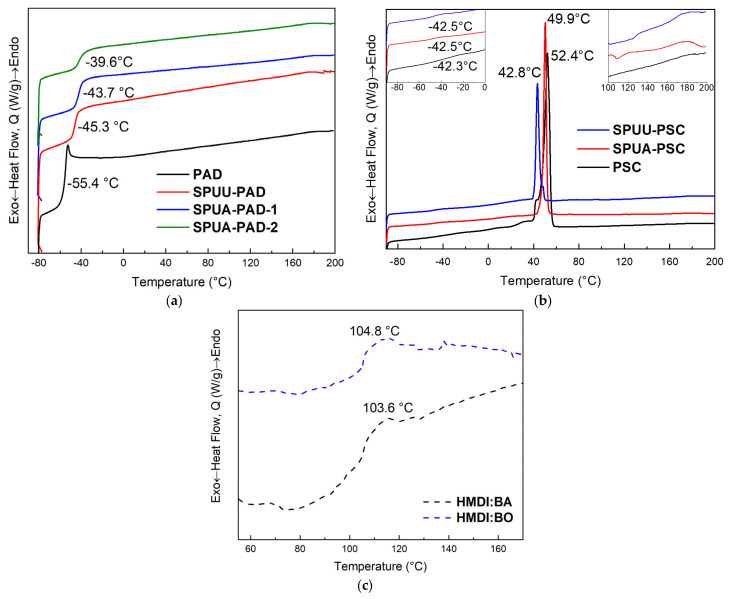
DSC thermograms of SPU synthesized with (**a**) PAD and (**b**) PSC, and (**c**) hard segments.

Differential scanning calorimetry characterization was performed to evaluate the microphase separation of SPU made with both polyols. Usually, in SPU with phase separation, up to four signals could be found, with two corresponding to the Tg and Tm of the polyol and the two others corresponding to the Tg and Tm of the hard segment. DSC thermograms of pristine PAD (Figure 8) show a glass transition temperature at −55.4 °C, whereas in SPUPAD, this event shifted to −45.3, −41.3, and −39.6 °C for SPUU-PAD, SPUA-PAD-1, and SPUA-PAD-2, respectively. Also, the heat flow variation at the Tg (∆Q) of PAD decreased with the incorporation of the hard segment and was further reduced as the hard-segment percentage increased. The shift in Tg was produced by a hard segment diluted in a soft-segment phase which slows down the mobility of the soft segment and results in an increase in glass transition temperature [32]. No hard-segment Tg was detected, thus indicating that it was dissolved in the soft-segment phase. In contrast, pristine PSC showed two endothermic events (see Figure 8b), a Tg at −42.3 °C and a Tm at 52.4 °C, while in their corresponding SPU, three characteristic endotherms were observed. The Tg of the polyol in the soft segment was identified to be −42.5 °C, with a Tm between 42.8 and 52.4 °C depending on composition, and finally, the Tg of the hard segment was close to 105 °C (see right inset in Figure 8b). The Tg values of the hard segment were confirmed by running DSC exclusively on them and are shown in Figure 8c. The three events in SPUPSC are an indication of phase separation, where SPUU-PSC shows a decreased Tm of pure PSC, suggesting a loss of crystallinity or that a fraction of the diluted hard segment reduces the size of the soft-segment crystals [33,34]. The absence of a melting peak for the hard segment (urethane or urea) could be due to the fact that HMDI is a mixture of isomers and does not crystallize, regardless of the chain extender (BO/BA) used.

To estimate the weight percentage of hard block impurities present in the soft phase, the Fox equation (Equation (4)) was applied according to the quantitative evaluation proposed in Reference [35].

(4) $$\frac{1}{T_{\mathrm{gmix}}}=\frac{M_{1}}{T_{g1}}+\frac{M_{2}}{T_{g2}}$$

where $M_{1}$ and $M_{1}$ are the weight fractions of soft and hard segments, and $T_{g1}$, $T_{g2}$, and $T_{g\mathrm{mix}}$ represent the Tg of the PAD, hard segment, and segmented polyurethane, respectively. The Tg of the hard segment ($T_{g2}$) was undetectable in the SPUPAD systems; therefore, this value (105 °C) was taken from the data obtained in the DSC in Figure 8c. Applying Equation (4), the weight fraction of the hard segment was 10.5%, 12%, and 16% in the SPUU-PAD, SPUA-PAD-1, and SPUA-PAD-2 systems, respectively. Moreover, assuming that the entire soft segment calculated stoichiometrically is available and forms a continuous phase, the hard block impurities present would be 76%, 88%, and 61.4% of the total hard segment in SPUU-PAD, SPUU-PAD-1, and SPUA-PAD-2, respectively. It is interesting to note that the chain extender affected the fraction of hard block impurities, likely because BA produces ureas that are capable of forming a higher number of hydrogen bonds than urethanes from BO. Also, if the hard segment increased, the phase mixing was reduced due to a lower amount of polyol.

### 3.3. Microstructure Analysis

**Figure 9:**
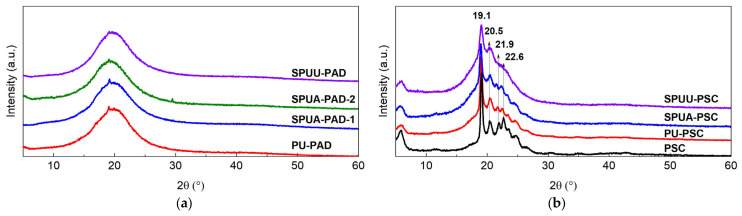
X-ray diffractograms of polyol/PU/SPUU/SPUA synthesized with (**a**) PAD and (**b**) PSC. Note: PAD diffractogram is not shown as it is a viscous liquid.

X-ray diffractograms, as depicted in Figure 9, confirmed that PU/SPUU/SPUA samples synthesized with PAD are completely amorphous, while those with PSC are semi-crystalline (θ = 19.1, 20.5, 21.9, and 22.6°). It is interesting to note that the polyol–isocyanate reaction as well as the incorporation of the hard segment did not induce a crystalline structure on the resulting materials. This result is in agreement with those observed in DSC thermograms (Figure 9a) and IR studies, where the urethane/urea carbonyl groups are hydrogen-bonded and disordered. Additionally, the diffractograms of SPUPSC show an amorphous halo attributed to the amorphous phase and less intense peaks from PSC as a result of the incorporation of HMDI and BO/BA. In addition, the degree of crystallinity of pristine PSC (22.8%) was reduced to 13.1%, 9.7%, and 8.1% for PU-PSC, SPUA-PSC, and SPUU-PSC, respectively.

DSC analysis of SPUU/SPUA synthesized with PAD showed the absence of a melting peak and that the Tg of polyol shifted to higher temperatures compared to that of pristine PAD, suggesting that there is a separate phase of soft segments that contains a small amount of hard segments. Also, it is known that the short hard segments of some SPUU/SPUA samples are dissolved in the soft segment due to the hydrogen bonding between the N-H (urethane/urea) and oxygen of the polyol [36], which limits the mobility of hard segment to separate and form a “pure” microphase [37] but results in mixed phases. Therefore, considering that PAD is a polyester triblock, there is the possibility that it can mix with the hard segment through hydrogen bonds. This observation is supported by the results obtained by IR studies, where urethane/urea carbonyl is predominantly hydrogen-bonded, likely resulting in a single-phase structure. This phenomenon impeded the identification/quantification of hard/soft segments using techniques such as DSC or TGA. Therefore, the low crystallization of the segments and the hydrogen bonds of the N-H groups probably helped in overcoming the incompatibility between phases, causing mixing between them. The majority of studies indicate that SPUA usually exhibits phase separation, but this was not observed in this study in polymers synthesized with PAD. This is consistent with the incomplete phase separation of hard segments of polyurethanes–ureas reported by Garrett et al. [38]. Additionally, it is expected that an increase in the hard segment rendered higher phase separation. This effect was not observed when the polymer composition changed from SPUA-PAD-1, with a hard-segment content of 13.4%, to SPUA-PAD-2, with hard-segment content of 23.6%. However, it has been reported that some copolymers with hard-segment percentages above 22% show a decrease in phase separation due to the hard segments being trapped in a nonequilibrium condition [39].

PSC-based SPUU/SPUA samples are semi-crystalline polymers with ordered regions, as shown in Figure 9b. This was confirmed by DSC thermograms, where phase separation was observed through the detection of a melting peak of the PSC, as well as two different Tg values and two thermal degradation events in TGA/DTGA, corresponding to the hard and soft segments. It is interesting to observe that in the SPUU-PSC sample, there was a greater reduction in diffractogram peak intensity and the largest decrease in the Tm value (from 52.4 °C to 42.8 °C). Therefore, it is likely that the HMDI:BO segment is highly amorphous [40] and diluted in the soft segment. In contrast, it is expected that the SPUA-PSC sample would produce ureas capable of forming a more ordered hard segment and a greater degree of phase separation, which could be related to the slight change in the Tm value (from 52.4 °C to 49.9 °C). This behavior is related to the observation that as the ordering becomes more effective, the degree of phase separation increases [34].

Thus, polyol is a crucial factor in enhancing phase separation, as SPUU/SPUA samples synthesized with PAD showed phase mixing, while those synthesized with PSC exhibited phase separation. This result can be attributed to the interaction between the soft and hard segments, as the more hydrogen bonds are formed by the polyol, the lower the degree of phase separation [41].

### 3.4. Degradation Studies

**Figure 10:**
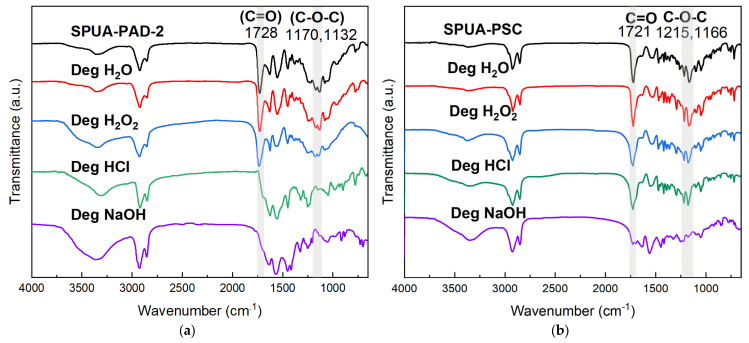
FTIR spectra of SPUA based on (**a**) PAD and (**b**) PSC after degradation in different media.

Degradation studies showed that oxidative, acidic, and alkaline media degrade both types of SPUA (PAD and PSC). SPUA-PAD-2 degraded by 5.5 ± 0.7%, 82.3 ± 8.0%, 70.8 ± 0.70%, and 50.0 ± 0.11% in $H_{2}O$, $H_{2}O_{2}$, HCl, and NaOH media, respectively, whereas SPUA-PSC showed a degradation of 2.2 ± 0.3%, 20.3 ± 1.2%, 45.5 ± 2.3%, and 67.9 ± 3.3% in $H_{2}O$, $H_{2}O_{2}$, HCl, and NaOH media, respectively. However, SPUA-PAD-2 samples are more prone to degradation as evidenced by the higher loss mass percentage observed and its FTIR spectra (Figure 10a) that showed the disappearance of bands related to C=O and C-O-C groups under hydrolytic degradations (HCl and NaOH), so it can be inferred that a large part of the flexible segment is degraded. It is interesting to note that the oxidative media ($H_{2}O_{2}$) caused more degradation on PAD-based polyurethane, but the corresponding spectra were similar to the non-degraded polyurethane, so it is likely that degradation occurred by surface erosion rather than in bulk or oligomer formation (chain scission) [42]. On the other hand, the IR spectra of the SPUA-PSC polymer (see Figure 10b) only showed C=O and C-O-C band disappearance in the alkaline medium (NaOH). It is interesting to observe that for both SPUAs, the OH peak around 3500 cm⁻¹ increased in the alkaline medium, suggesting that PAD and PSC were hydrolyzed, forming an OH group.

### 3.5. Mechanical Behavior

#### 3.5.1. Uniaxial Tension Test on SPUU/SPUA Films

**Figure 11:**
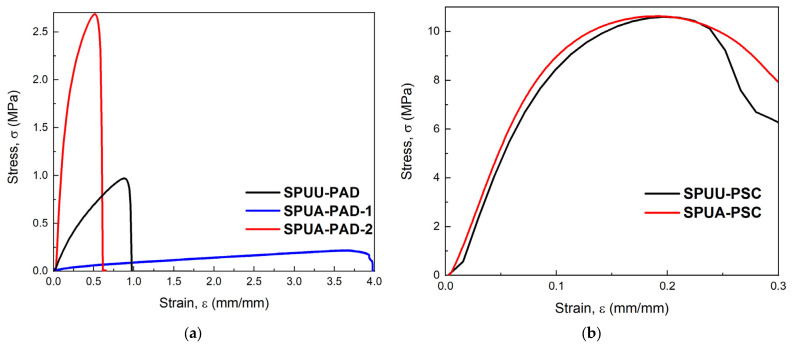
Representative uniaxial tension stress–strain curves of SPUU/SPUA films synthesized with (**a**) PAD and (**b**) PSC.

The PU-PAD sample did not form a film, while PU-PSC was a fragile and brittle polymer. Therefore, these PU models were discarded for mechanical studies and for use in the manufacture of nerve guide conduits. The mechanical properties of the films of the synthesized SPUU/SPUA are reported in Table 2, and stress–deformation curves are shown in Figure 11.

SPUPAD films exhibited a marked difference in their mechanical properties. When butanediol was used as a chain extender (SPUU-PAD), it showed a higher mechanical strength and Young’s modulus (E) than when butanediamine was used in the chain extension reaction (SPUA-PAD-1) despite the fact that 1,4 butanediamine can yield ureas that are capable of forming more hydrogen bonds in comparison with 1,4 butanodiol, which produces urethanes. In SPUA-PAD-1 with ureas, a maximum deformation of 3.91 ± 0.12 mm/mm was achieved, probably due to the higher mixing phase, where mechanical properties are governed by the elasticity and deformation of soft segments. In contrast, SPUU-PAD with urethane groups in the hard segment produced higher stiffness but lower deformation. Furthermore, when the hard-segment content was increased from 13.4% to 23.6% as in SPUA-PAD-2, it caused an increase in stiffness and mechanical strength, suggesting that the presence of physical cross-links acts as a filler that reinforces the soft segment [43]. Also, when the hard segment increased, it caused enhanced mechanical behavior in these SPUs.

In SPUPSC films the chain extender effect was the opposite. SPUU-PSC showed mechanical properties slightly lower than SPUA-PSC. In these polymers, hard and soft segments are separated, and usually, when stress is applied, the soft segment is deformed elastically and then loads are transferred to the hard segment [44]. Polyurethanes–ureas are characterized by stronger interactions, generally resulting in stiffer materials due to N-H bonding in the hard domain [45]. Thus, it is expected that in polymers made with PSC, segmented polyurethane–urea has better mechanical properties.

Stiffness is an important factor that regulates axonal regeneration and affects cell adhesion, differentiation, and migration [46]. In addition, neuron and Schwann cell myelination is regulated by mechanotransduction signaling; thus, an appropriate biomechanical microenvironment is required [47]. Mosley et al. reported that a Young’s modulus of 907 kPa (closer to the modulus of the brain and spinal cord) allowed axonal extensions [48]. Therefore, the mechanical properties of nerve conduits are important parameters to achieve peripheral nerve repair. Although many of the chemical requirements in nerve regeneration have been studied, mechanical stiffness is a parameter that has not been elucidated. In this study, Young’s modulus, mechanical strength, and maximum deformation values of peripheral nerve tissues were used as a parameter to assess the behavior of the segmented polyurethanes and polyurethanes–ureas synthesized in our study. Dumon and Born [49] reported an E of 15.87 ± 2.21 and 8.19 ± 7.27 MPa, $\sigma_{\max}$ of 6.78 ± 0.57 and 8.54 ± 3.3 MPa, and $\epsilon_{\max}$ 0.61 ± 0.02 and 1.64 ± 0.34 mm/mm for intact and extracted human nerves, respectively.

**Table 2:** Mechanical properties. Uniaxial tension test on SPUU/SPUA films prepared with PAD and PSC and longitudinal tension test on NGC-SPUA-PAD-2 (mean value ± SD, n = 6), and their comparison with native nerves and other NGCs reported.

Structure	$F_{\max}$ (N)	$\delta_{\max}$ (mm)	E (MPa)	$\sigma_{\max}$ (MPa)	$\epsilon_{\max}$ (mm/mm)
SPU Films					
SPUU-PSC	4.62 ± 1.21	2.25 ± 0.12	107 ± 5	10.3 ± 0.2	0.18 ± 0.01
SPUA-PSC	5.29 ± 0.53	2.31 ± 0.22	117 ± 7	10.8 ± 0.3	0.22 ± 0.04
SPUU-PAD	0.88 ± 0.08	10.1 ± 0.7	1.87 ± 0.06	0.95 ± 0.06	0.84 ± 0.06
SPUA-PAD-1	0.18 ± 0.01	43.4 ± 0.1	0.10 ± 0.01	0.20 ± 0.01	3.91 ± 0.12
SPUA-PAD-2	0.90 ± 0.24	5.07 ± 1.54	16.3 ± 2.2	2.14 ± 0.14	0.42 ± 0.06
NGC					
NGC-SPUA-PAD-2	21.8 ± 2.3	14.3 ± 3.9	8.84 ± 3.8	1.83 ± 0.27	0.45 ± 0.13
NGC-SPUA-PAD-2-IN **	11.1 ± 1.6	9.31 ± 1.47	4.46 ± 0.5	0.76 ± 0.11	0.31 ± 0.05
Intact Nerve [49]	19.85 ± 7.21	-	15.87 ± 2.21	6.78 ± 0.57	0.61 ± 0.02
Extracted Nerve [49]	33.56 ± 6.07	-	8.19 ± 7.27	8.54 ± 3.30	1.64 ± 0.34
NeuraGen^®^ Conduit ** [50]	6.89 ± 2.6	-	-	-	-
NeuroTube^®^ Conduit [51]	-	-	4 ± 2	13 ± 3	2.76 ± 46
SilkBridgeTM Conduit * [52]	26.7 ± 2.3	-	3.3 ± 0.6	-	0.75 ± 0.06
PU NGC * [51]	-	-	6 ± 1	2	2.55 ± 0.11
PU NGC ** [53]	4.98 ± 0.35	-	-	6.37 ± 0.5	-

* Wall thickness ~0.5 mm; ** mechanical properties after conditioning.

Considering this, SPUU/SPUA films based on PSC exhibited a modulus of 107 ± 5 and 117 ± 7 MPa (SPUU-PSC and SPUA-PSC, respectively) which is one order of magnitude higher than the human nerve; i.e., this property is exceeded and this modulus mismatch could interfere with proper nerve repair. However, the formulation with the mechanical properties closest to the peripheral nerve was SPUA-PAD-2, which exhibited a modulus of 16.3 ± 2.2 MPa, mechanical strength of 2.14 ± 0.14 MPa, and maximum deformation of 0.42 ± 0.06 mm/mm. However, it should be noted that these mechanical tests were conducted on films, whereas nerve conduits are considered tubular structures. For this reason, the polyurethane–urea formulation was selected for manufacturing tubes (conduits), which were then characterized mechanically through longitudinal and radial tension tests.

#### 3.5.2. Longitudinal and Radial Tension Tests on SPUA-PAD-2 Conduits

In order to determine the mechanical properties of the tubes and rings under usage conditions, samples were conditioned in distilled water at 25 °C for 30 days. After the incubation time, the wet tubes and rings were placed in the universal testing machine to perform radial and longitudinal tension tests.

**Figure 12:**
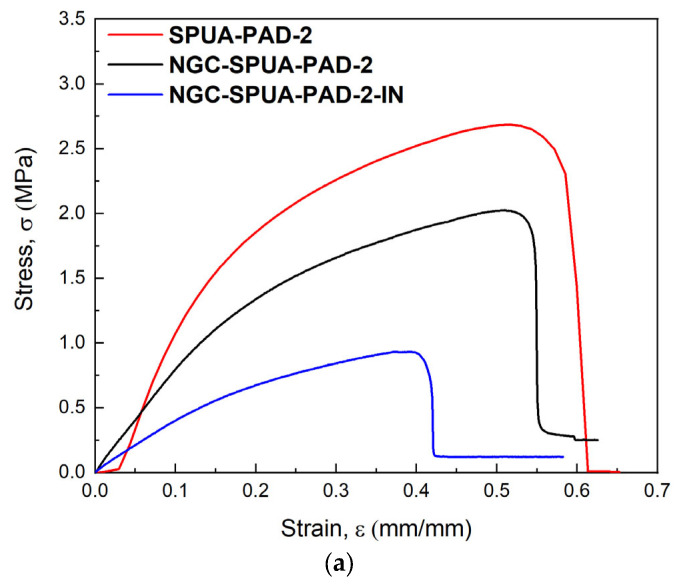

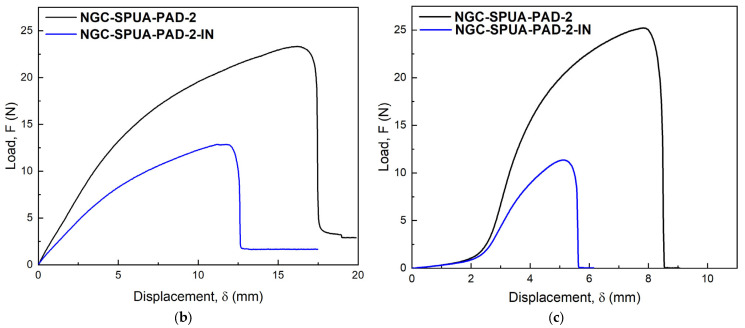
Representative curves of NGC-SPUA-PAD-2 and NGC-SPUA-PAD-2-IN: (**a**) strain–stress, (**b**) longitudinal tension load–displacement, and (**c**) radial tension load–displacement.

Nerve conduits made of SPUA-PAD-2 polyurethane–urea were labeled as NGC-SPUA-PAD-2. The mechanical properties in the longitudinal tension test of NGC-SPUA-PAD-2, non-conditioned and incubated (NGC-SPUA-PAD-2-IN), are listed in Table 2, and representative curves are shown in Figure 12. The NGC-SPUA-PAD-2 properties in Table 2 were compared to those of SPUA-PAD-2 films, native nerves, FDA-approved NGCs (NeuraGen^®^ and NeuroTube^®^), and NGCs proposed in other studies.

When comparing SPUA-PAD-2 films and NGC-SPUAPAD (Figure 12a) in terms of longitudinal tension, it was found that the Young’s modulus (which is an intrinsic material property) and mechanical strength values were reduced in the tubular geometry. In the films, the values of E = 16.3 ± 2.2 and $\sigma_{\max}$ = 14 ± 0.14 MPa decreased for NGC-SPUA-PAD-2 to E = 8.84 ± 3.81 MPa and $\sigma_{\max}$ = 1.83 ± 0.27 MPa for non-conditioned conduits. This effect may be due to imperfections in the guidance channels, such as the presence of microbubbles created by solvent evaporation during manufacture. In the case of maximum deformation, the values for the films and conduits were similar (0.42 ± 0.06 and 0.45 ± 0.13 mm/mm, respectively). Furthermore, it can be observed in Figure 12b that the mechanical properties of NGC-SPUA-PAD-2 decreased even more when it was conditioned in an aqueous medium. Under conditioning, the elastic modulus and maximum stress of the incubated conduit decreased to 4.46 ± 0.5 and 0.76 ± 0.11 MPa, respectively. This reduction of nearly half in the mechanical properties may be due to swelling, which increased the conduit’s thickness by 4% with a total weight gain of 5%, as measured gravimetrically.

The representative force–displacement curve for the radial tensile test of the NGC-SPUA-PAD-2 rings is shown in Figure 12c. The maximum radial force calculated for the dry tubes was 25.1 ± 1.24 N and the circumferential tensile strength was 1.32 ± 0.06 N/mm, while for the conditioned tubes, these values were 11.24 ± 2.03 N and 0.56 ± 0.11 N/mm, respectively. As in the case of the longitudinal tension tests, the radial tension mechanical properties also decreased by more than half.

It is desirable that the mechanical properties of NGCs match the stiffness, flexibility, and elongation of native nerves to achieve more effective therapeutic outcomes. Additionally, the NGC-SPUA-PAD-2-IN fabricated in this work had modulus, mechanical strength, and maximum deformation values, when conditioned, within the range of values reported by Philips et al. [1] and Mankavi et al. [54] for peripheral nerves.

It was observed that the moduli of NGC-SPUA-PAD-2 (8.84 ± 3.8 MPa) and NGC-SPUA-PAD-2- IN (4.46 ± 0.5 MPa) tubes were lower than those of native intact nerves (15.87 ± 2.21 MPa) but similar to those of extracted nerves (8.19 ± 7.27 MPa), as reported by Dumont and Born [49]. In contrast, mechanical strength exhibited a pronounced difference, as nerves had higher ultimate stress compared to NGC-PAD. However, this parameter indicates the stress at which nerve failure occurs; therefore, it is not necessary for an NGC to withstand this level of stress. Additionally, the maximum strain of native nerves (0.61 ± 0.02 mm/mm) and the extracted-nerve ECM (1.64 ± 0.34 mm/mm) is superior to that of NGC-SPUA-PAD-2 (0.45 ± 0.13 mm/mm) and NGC-SPUA-PAD-2-IN (0.31 ± 0.05 mm/mm). Nevertheless, it is not necessary for the NGCs to be deformed to these values because nerves elongate only by 6 to 8% during normal body movement [54]. With respect to commercial NGCs, NGC-SPUA-PAD-2 (maximum load = 21.8 ± 2.3 N) exhibited higher mechanical properties than those reported for the collagen-based NeuraGen^®^ conduit (maximum load = 6.89 ± 2.6 N) [50]. The SilkBridgeTM conduit (with a wall thickness of 0.52 mm) and NGC-SPUA-PAD-2 exhibited similar mechanical properties, but NeuroTube^®^ exhibited a similar modulus to NGC-SPUA-PAD-2-IN (incubated tube). Hsu et al. [51] reported significant efficacy in nerve regeneration using a PU NGC based on PCL and poly (ethylene butylene adipate), with a modulus and mechanical strength close to the NGC-SPUA-PAD-2 manufactured in this study. In contrast, in the wet state, Niu et al. reported a maximum load of 4.98 ± 0.35 N for PU NGCs based on PCL and PEG; thus, NGC-SPUA-PAD-2-IN exhibited a greater value of 11.1 ± 1.6 N.

## 4. Conclusions

IR and Raman spectroscopy studies consistently showed the presence of urethane and urea linkages as a result of the reaction between adipate/sebacate polyols–HMDI and HMDI-BO/BA, indicating that SPU/SPUA synthesis was successful. In agreement with this, 1H NMR analysis confirmed the chemical structure of both polyols and the formation of urethanes/urea in segmented polyurethanes by the identification of the NH proton signal. X-ray diffractograms showed that PAD and PSC were amorphous and semi-crystalline polymers, respectively, while DSC and TGA studies demonstrated phase mixing when PAD was used as a polyol and phase separation when PSC was used within the polyurethane structure. In semi-crystalline SPUPSC films, the incorporation of 1,4-butanediamine promoted better mechanical properties due to stronger interactions (N-H bonding) and higher phase separation compared to 1,4-butanediol. In contrast, in amorphous SPUPAD films, 1,4-butanediamine was capable of further mixing the phases, lowering their mechanical properties. Measured mechanical properties allowed the use of SPUA-PAD-2 for manufacturing nerve guidance conduits (NGC-SPUA-PAD-2), which exhibited a modulus nearly identical to that of native nerves in longitudinal tests and after water conditioning. The long-term performance of these nerve conduits also depends on their degradation, as a significant portion of the flexible segment is degraded under hydrolytic conditions. However, this can be further exploited in tissue engineering where the scaffold is degraded as the extracellular matrix is developed.

## Figures and Tables

**Table 1:** Hard- and soft-segment percentages in synthesized SPUU/SPUA.

Designation	Composition *	SS %	HS %
PU-PAD	PAD:HMDI (1:1)	100	0
SPUU-PAD	PAD:HMDI:BO (1:2:1)	86.6	13.4
SPUA-PAD-1	PAD:HMDI:BA (1:2:1)	86.6	13.4
SPUA-PAD-2	PAD:HMDI:BA (1:3:2)	76.4	23.6
PU-PSC	PSC:HMDI (1:1)	100	0
SPUU-PSC	PSC:HMDI:BO (1:2:1)	88.1	11.9
SPUA-PSC	PSC:HMDI:BA (1:2:1)	88.1	11.9

* Molar ratio= polyol–HMDI–chain extender.

## Data Availability

The data presented in this study are available upon request from the corresponding author.
